# Risky sexual behaviors among orphaned youth in South Africa: findings of the 2017 population-based household survey

**DOI:** 10.3389/frcha.2023.1033663

**Published:** 2023-10-20

**Authors:** Noloyiso Vondo, Musawenkosi Mabaso, Vuyelwa Mehlomakulu, Ronel Sewpaul, Adlai Davids, Philisiwe Ndlovu, Derrick Sekgala, Londiwe Shandu, Sizulu Moyo

**Affiliations:** ^1^ Human and Social Capabilities, Human Sciences Research Council, Cape Town, South Africa; ^2^ Faculty of Health Sciences, Nelson Mandela University, Port Elizabeth, South Africa; ^3^ UCT School of Public Health and Family Medicine, University of Cape Town, Cape Town, South Africa

**Keywords:** orphanhood status, youth, risky sexual behavior, household survey, South Africa

## Abstract

**Introduction:**

In sub-Saharan Africa, evidence shows that orphaned youth are exposed to more risky sexual behaviors than non-orphaned peers, which increases the chances of contracting HIV and other sexually transmitted infections. The fast rises in the prevalence of orphanhood are a result of the HIV/AIDS epidemic.

**Methodology:**

The data for this secondary analysis were collected through a cross-sectional, multi-stage, stratified, cluster randomized sampling design. Multivariable backward stepwise logistic regression analysis was fitted to determine the factors associated with risky sexual behaviors among male and female adolescents and youth aged 12–24 years.

**Results:**

Of 2,556 orphaned participants, 18.3% (95% CI: 14.3–23.0) had two or more sexual partners in the last 12 months, 31.3% (95% CI: 26.3–36.6) reported no condom use at last sex, and 98.3% (95% CI: 96.6–99.2) reported no consistent condom use. The likelihood of reporting multiple sexual partnerships was significantly lower among female adolescents, those residing in rural formal/farm areas, and those who reported sexual debut at age 15 years and was higher among those who reported sexual partners 5 years and older than their age group. The odds of not using a condom at last sex were significantly higher among female adolescents and those who perceived themselves as being at high risk of HIV infection and were significantly lower among those who had sexual debut at age 15 years and older.

**Discussion:**

The findings suggest that there is a need for sexual risk-reduction strategies targeted at orphans, especially male orphans and those residing in urban areas. Such efforts should include behavior change interventions for delaying the age of sexual debut, changing HIV risk perception, mitigating multiple sexual partnerships, age-disparate sexual relationships, and enhancing condom use.

## Introduction

Risky sexual behaviors among orphans remain a major public health concern, especially in sub-Saharan Africa (1). Orphans are defined as children aged under 18 years whose mother, father, or both parents have died (2). A child whose mother has died is known as a maternal orphan, a child whose father has died is a paternal orphan, and a child who has lost both parents is a double orphan. According to Statistics South Africa, there were 2.7 million orphans in 2018 (3). In South Africa, many adolescents and youth were orphaned as children at the height of the HIV epidemic before the scaling up of antiretroviral treatment (4, 5).

In sub-Saharan Africa, evidence shows that orphaned children and youth are exposed to more risky sexual behaviors than their peers, possibly due to decreased parental oversight and support (2). In addition, orphans often fall prey to sexual coercion, exploitation, and abuse and may be forced to engage in high-risk behaviors (6, 7). These behaviors include unprotected sexual intercourse, early sexual initiation, having multiple sexual partners, age-disparate sexual relationships, and transactional sex in exchange for money, goods, or other favors (2, 6, 7). Early sexual activity in this population group can also be partly due to the desire for closeness and security, which leads to HIV-related risky behavior that maintains high levels of HIV infection in this country (8, 9).

Risky sexual behaviors increase susceptibility to sexually transmitted infections, including HIV, and unplanned or unwanted pregnancies, which in turn can lead to deleterious health, social, and economic consequences (8–10). The vulnerability of orphans to sexual risk behaviors have been attributed to socio-demographic (such as being younger, gender disparities, low educational attainment, and poverty) and socio-behavioral factors (such as substance use, peer influence, sexual predation, and lack of family support) (8, 11). These culminate in high-risk abortion, psychological disorders, teenage pregnancy, school dropout, and early marriage (2).

In South Africa, several programs have been implemented to reduce vulnerability and/or protect orphaned children and youth. These include traditional institutional care, as well as institutional programs that are embedded within community- and/or family-based care (12, 13). Community-based care is a measure aimed at allowing children to stay with their relatives or review extended families to avoid separation and/or placement with an alternate family (12, 13). The community-based approach has the advantage of allowing children to be cared for by familiar individuals while remaining in their local communities. Furthermore, family-based care is more likely to satisfy their developmental needs and provide them with relevant information and skills they will need to live independently in their communities (14).

However, others also found that orphans in community- and/or family-based care are exposed to the prevailing community pressures and norms and may be more vulnerable to sexual coercion, exploitation, and abuse, and therefore more prone to risky sexual behaviors depending on the setting and context (7, 15, 16). Understanding the magnitude of risky sexual behaviors and exploring predisposing factors is critical for addressing the challenges of orphans to mitigate negative health outcomes. Therefore, the purpose of this study was to examine the factors associated with risky sexual behaviors among orphaned adolescents and youth aged 12–24 years in South Africa using data from the 2017 nationally representative household-based population survey.

## Methodology

### Data source

The data used in this analysis are from the 2017 nationally representative population-based HIV household survey conducted in 2017 using a multi-stage, stratified, random cluster sampling design described in detail elsewhere (17). Basically, a total of 1,000 small area layers (SALs) were drawn from a national master sampling frame of 86,000 SALs released by Statistics South Africa in 2001 and updated in 2011 (18). The selection of SALs was stratified by province, locality type (urban areas, rural informal and formal areas), race group, and sex. A systematic probability sample of 15 visiting points per household was drawn from each of the 1,000 SALs yielding a total of 15,000 visiting points per household targeted for the survey.

Survey data were collected using a household questionnaire and three age-appropriate questionnaires, which were administered to consenting individuals. For those aged younger than 18 years, consent was given by parents/guardians and assent by the participants aged 12–18 years. The interview instruments solicited information on socio-demographic characteristics, including orphanhood status, sexual behaviors, HIV knowledge and risk perceptions, and health-related factors including HIV testing. The questionnaires were administered by fieldworkers and electronically captured using tablets. Fieldworkers also collected dried blood spot samples from participants using a finger prick for HIV biomarker testing (HIV antibodies, exposure to antiretrovirals (ARVs), and lag testing for incidence estimation).

### Measures

The orphanhood status variable was based on two questions: Is your biological mother alive? (yes or no) and Is your biological father alive? (yes or no). Responses were pooled and dichotomized into a binary outcome (yes = 1, no = 0) indicating orphanhood status among adolescents and youth in the review aged 12–24 years. Three primary outcome variables included: (1) multiple sexual partners based on the question: How many sexual partners did you have in the last 12 months? (one partner = 0, two or more partners = 1); (2) no condom use at last sex act based on the question: Did you use a condom at last sexual encounter with the most recent person you had sex with? (no = 1, yes = 0); and (3) inconsistent condom use based on the question: How often do you use a condom with your most recent sexual partner? Responses were dichotomized into a binary outcome (1 = every time, 0 = almost every time, 0 = sometimes, and never = 0).

The explanatory variables included socio-demographic, socio-behavioral, and HIV-related factors. The socio-demographic factors included sex (male and female), age (12–19 years, 20–24 years), race (Black African and other, including White, Colored, and Indian), highest level of education attained (no education or up to primary, secondary, and tertiary), and asset-based socioeconomic status (SES) score. A composite measure based on the availability of essential services and ownership of a range of household assets was used to compute five wealth quintiles (first lowest, second lower, third middle, fourth higher, and fifth highest) representing a continuum of household SES from the poorest to the least poor. The quintiles were then dichotomized into low SES or poorest (the three lowest quintiles) and high SES or less poor (the two highest quintiles). Locality type (urban, rural informal/tribal area, and rural formal/farm areas) and disability (yes, no) were also included.

Socio-behavioral factors included the following: ever had sexual intercourse (no, yes); age of sexual debut (had sex before the age of 15 years, had sex at age15 years and older); age of sexual partner (partner more than 5 years younger, partner within 5 years of age, partner more than 5 years older); number of sexual partners in the last 12 months (one partner, two or more partners); condom use at last sex (yes, no); alcohol use using the Alcohol Abuse Disorder Identification Test (AUDIT) score [abstainers, low risk (with scores in the range of 1–7), risky/hazardous level (8–15), high risk/harmful (16–19), very high risk (20+)]. HIV-related factors included the following: correct knowledge and rejection of all myths about HIV transmission (yes, no); self-perceived risk of HIV infection (yes, no); ever tested for HIV (yes, no); awareness of HIV status (yes, no); and the survey laboratory results determined an HIV status (HIV positive, HIV negative).

### Ethical approval

The survey protocol was approved by the Human Sciences Research Council (HSRC) Research Ethics Committee (REC: 4/18/11/15) and both the Division of Global HIV and TB (DGHT) and the Center for Global Health (CHG) of the Centers for Disease Control and Prevention (CDC). Verbal or written informed consent was sought before undertaking both the behavioral data and blood specimen collections. The confidentiality and anonymity of the participants were also assured.

### Statistical analysis

Descriptive statistics were used to summarize the characteristics of the study sample. Cross-tabulations and chi-square (χ²) tests were used for the comparison of categorical background characteristics, risky sexual behaviors, and HIV-related variables by orphanhood status. A multivariable stepwise backward logistic regression analysis was used to determine the factors associated with orphan status by fitting a male and female model. The probability for the removal of variables in the models was set at *p*-values of 0.2. Adjusted odds ratio (AOR) with 95% confidence intervals (CI) and a *p*-value ≤ 0.05 were used to determine the level of statistical significance. Sample weights were introduced to all models to account for the complex survey design and non-response. Statistical analyses were carried out using Stata statistical software, Release 15.0 (Stata Corporation, College Station, TX, USA). Coefficient plots were used to display the results of the final models.

## Results

### Sample characteristics

**Figure 1:**
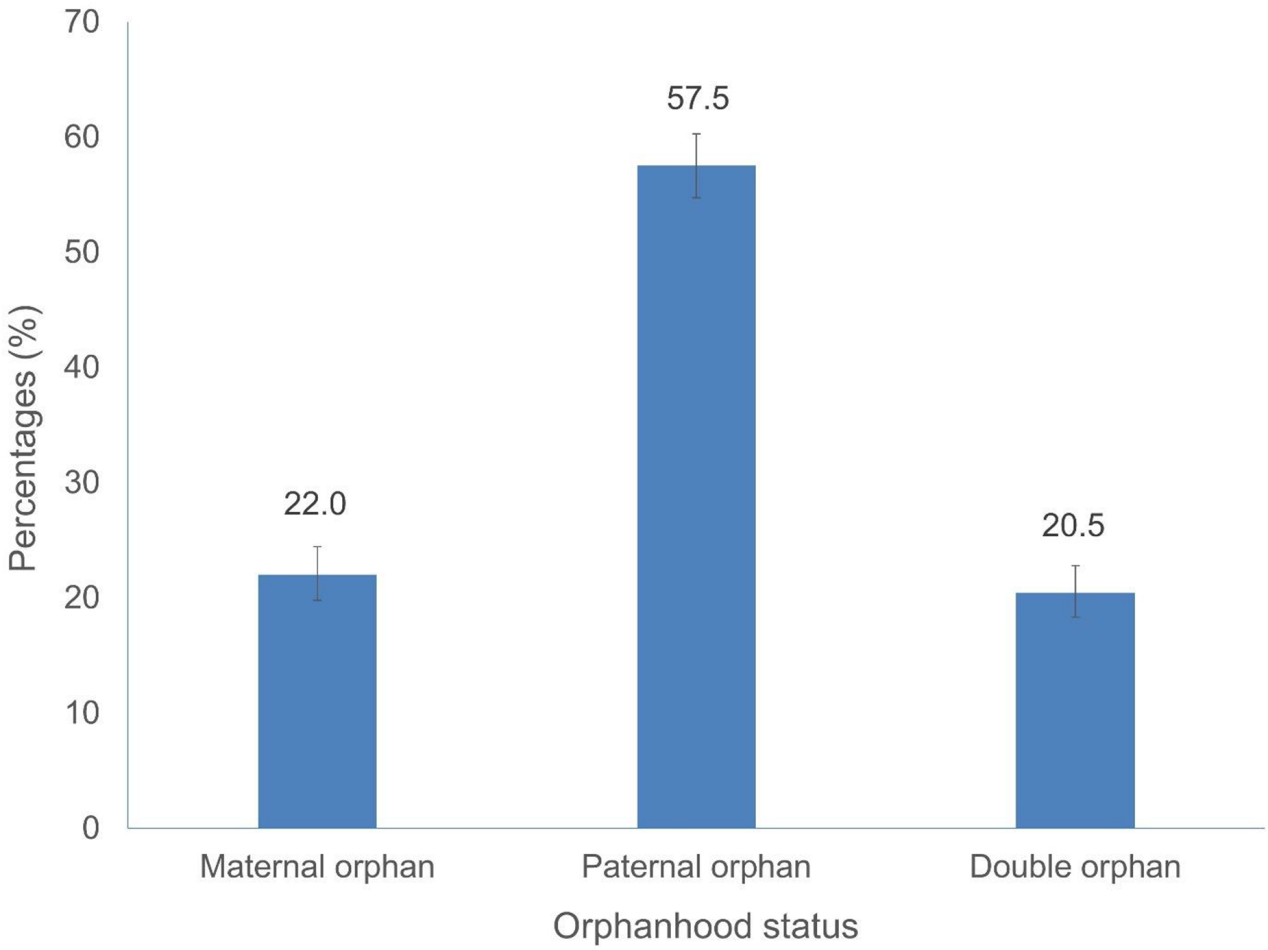
Orphanhood status among adolescents and youth aged 12–24 years, South Africa 2017.

Figure 1 shows the orphanhood status of the study sample. A higher proportion of study participants were paternal orphans (57.5%).

Table 1 shows the background characteristics of the study sample. Of the 2,556 orphaned participants, the majority were aged 12–19 years (83.5%). The mean age was 17.7 years (SD = 3.7). Half of the study sample were male participants (51.2%). Most participants were African (92.0%), had secondary-level education (84.5%), and were unemployed (95.7%). More than half of the participants were from high SES households (54.6%) and urban areas (53.8%). Most participants stated that they had a sexual debut at age 15 years and older (92.8%), had sexual partners within 5 years of their age group (79.3%), and abstained from alcohol (86.5%). In addition, most participants had correct knowledge of HIV and rejected myths about HIV (69.8%) and had a low self-perceived risk of HIV infection (85.9%). Of the participants, 45.6% reported that they had ever tested for HIV and approximately 33.8% reported that they were aware of their HIV status. Overall, 7.7% of the study participants were HIV positive.

**Table 1:** Characteristics of the study sample (*n* = 2,556), adolescents and youth aged 12–24 years, South Africa 2017 survey.

Variables	Total	%	95% CI
Age categories			
12–19	2,175	83.5	81.3–85.5
20–24	381	16.5	14.5–18.7
Sex			
Male	1,162	48.8	46.1–51.5
Female	1,394	51.2	48.5–53.9
Race groups			
African	2,306	92.0	90.5–93.2
Other	250	8.0	6.8–9.5
Education level attained			
No education/primary	63	11.5	8.5–15.5
Secondary	427	84.5	79.9–88.3
Tertiary	20	3.9	2.0–7.5
Employment status			
Not employed	1,737	95.7	94.2–96.8
Employed	87	4.3	3.2–5.8
Asset-based SES			
Low SES	1,320	54.6	51.7–57.4
High SES	981	45.4	42.6–48.3
Locality type			
Urban	1,160	53.8	51.2–56.5
Rural informal (tribal areas)	1,199	42.9	40.3–45.5
Rural (farms)	197	3.3	2.7–4.0
Age at sexual debut			
Less than 15 years	119	7.2	5.7–9.0
15 years and older	1,718	92.8	91.0–94.3
Age-disparate sexual relationship			
Within 5 years	438	79.3	74.5–83.4
Younger than 5 years	4	1.2	0.4–3.5
Older than 5 years	125	19.6	15.6–24.2
AUDIT score			
Abstainers	2,128	86.5	84.3–88.4
Low risk drinkers (1–7)	183	9.1	7.5–11.0
High risk drinkers (8–19)	74	4.2	3.1–5.5
Hazardous drinkers (20+)	8	0.2	0.1–0.6
Correct knowledge of HIV and myth rejection			
No	1,748	69.8	67.3–72.3
Yes	798	30.2	27.7–32.7
Self-perceived risk of HIV infection			
Low	2,135	85.9	83.9–87.7
High	332	14.1	12.3–16.1
Ever had an HIV test			
Yes	1,138	45.6	42.9–48.3
No	1,352	54.4	51.7–57.1
Awareness of HIV status			
Yes	848	33.8	31.2–36.5
No	1,649	66.2	63.5–68.8
Final HIV status			
Positive	165	7.7	6.3–9.5
Negative	1,813	92.3	90.5–93.7

Subtotals do not equal to total (*n*) due to non-response and/or missing data.

### Sample characteristics and risky sexual behaviors

Of the 2,556 orphaned participants, 18.3% (95% CI: 14.3–23.0) who had two or more sexual partners in the last 12 months, 31.3% (95% CI: 26.3–36.6) reported no condom use at last sex and 98.3% (95% CI: 96.6–99.2) reported no consistent condom use. Table 2 shows the distribution of risky sexual behaviors by sample characteristics. Having multiple sexual partners was significantly higher among adolescents (12–19 years), female participants, those who reported sexual debut before 15 years of age, those who perceived themselves as being at high risk of HIV infection, and those who were not aware of their HIV status. No condom use at last sex was significantly higher among female participants, other race groups, those with sexual partners older than 5 years of their age, those who perceived themselves as being at high risk of HIV infection, and those who were HIV positive. Inconsistent condom use was significantly higher among female participants, those not employed, those who had sexual debut at 15 years of age, those with partners within 5 years of their age, those who had correct knowledge and rejected myths about HIV, and those who were aware of their HIV status.

**Table 2:** Multiple sexual partners, condom use at last sex and consistent condom use among adolescents and youth aged 12–24 years, South Africa 2017 survey.

Variables	Number of sexual partners in the last 12 months						Condom use last sex act						Consistent condom use					
	*N*	One partner		Two and more partners			*N*	No		Yes			*N*	No		Yes		*p*-value
		%	95% CI	%	95% CI	*p*-value		%	95% CI	%	95% CI	*p*-value		%	95% CI	%	95% CI	
Total	573	81.7	77.0–85.7	18.3	14.3–23.0		580	31.3	26.3–36.6	68.7	63.4–73.7		577	98.3	96.6–99.2	1.70	0.8–3.4	
Age groups																		
12–19 years	365	77.9	71.2–83.4	22.1	16.6–28.8	0.028	373	27.2	21.6–33.7	72.8	66.3–78.4	0.057	370	98.7	96.0–99.6	1.30	0.4–4.0	0.420
20–24	208	87.5	80.8–92.2	12.5	7.8–19.2		207	37.6	29.1–47.0	62.4	53.0–70.9		207	97.7	94.6–99.0	2.30	1.0–5.4	
Sex																		
Male	241	73	65.2–79.6	27	20.4–34.8	<0.001	245	22.2	15.9–30.2	77.8	69.8–84.1	0.001	242	96.5	93.0–98.3	3.50	1.7–7.0	0.004
Female	332	89.6	83.5–93.6	10.4	6.4–16.5		335	39.7	32.6–47.2	60.3	52.8–67.4		335	100.0		0.00		
Race groups																		
African	529	81.7	76.8–85.8	18.3	14.2–23.2	0.992	536	30.3	25.3–35.8	69.7	64.2–74.7	0.030	533	98.4	96.6–99.2	1.60	0.8–3.4	0.353
Other	44	81.7	65.9–91.1	18.3	8.9–34.1		44	51	32.6–69.2	49	30.8–67.4		44	96.6	87.1–99.2	3.40	0.8–12.9	
Education level attained																		
No education/primary	21	79.8	47.2–94.6	20.2	5.4–52.8	0.868	20	68.8	41.2–87.4	31.2	12.6–58.8	0.365	21	100.0		0.00		0.871
Secondary	229	80.1	71.9–86.3	19.9	13.7–28.1		228	43.8	34.9–53.1	56.2	46.9–65.1		227	98.2	94.7–99.4	1.80	0.6–5.3	
Tertiary	7	88.5	43.2–98.7	11.5	1.3–56.8		7	45.6	8.9–87.8	54.4	12.2–91.1		7	100.0		0.00		
Employment status																		
Not employed	522	81.7	76.7–85.9	18.3	14.1–23.3	0.793	521	31.8	26.6–37.5	68.2	62.5–73.4	0.497	518	98.6	96.9–99.4	1.40	0.6–3.1	0.026
Employed	39	83.7	65.5–93.3	16.3	6.7–34.5		38	24.7	10.9–46.7	75.3	53.3–89.1		37	92.2	71.0–98.3	7.80	1.7–29.0	
Asset-based SES																		
Low SES	299	81.6	74.8–86.9	18.4	13.1–25.2	0.766	305	33.1	26.5–40.4	66.9	59.6–73.5	0.771	304	98.1	94.9–99.3	1.90	0.7–5.1	0.933
High SES	223	80.2	72.1–86.4	19.8	13.6–27.9		224	31.4	23.4–40.6	68.6	59.4–76.6		222	98.2	95.3–99.3	1.80	0.7–4.7	
Locality type																		
Urban areas	290	80.6	73.8–86.0	19.4	14.0–26.2	0.280	297	30	23.4–37.5	70	62.5–76.6	0.460	296	97.9	95.5–99.1	2.10	0.9–4.5	0.709
Rural informal (tribal areas)	237	82.4	75.1–87.9	17.6	12.1–24.9		239	32.3	25.3–40.2	67.7	59.8–74.7		237	98.8	94.6–99.7	1.20	0.3–5.4	
Rural (farms)	46	95.4	83.9–98.8	4.6	1.2–16.1		44	44.4	26.4–64.1	55.6	35.9–73.6		44	100.0		0.00		
Age at sexual debut																		
Less than 15 years	79	59.9	45.4–72.8	40.1	27.2–54.6	<0.001	80	40.4	27.5–54.8	59.6	45.2–72.5	0.127	253	91.2	84.9–95.0	8.8	5.0–15.1	0.013
15 years and older	489	85.2	80.2–89.0	14.8	11.0–19.8		486	29.3	24.0–35.1	70.7	64.9–76.0		1,397	98.8	97.8–99.3	1.2	0.7–2.2	
Age-disparate relationship																		
Within 5 years	433	82.8	77.5–87.0	17.2	13.0–22.5	0.322	433	27.7	22.3–33.9	72.3	66.1–77.7	0.001	1,286	97.5	96.0–98.4	2.5	1.6–4.0	<0.001
Younger than 5 years	4	51.2	10.2–90.7	48.8	9.3–89.8		4	6.8	0.7–41.9	93.2	58.1–99.3		9	80.3	31.8–97.3	19.7	2.7–68.2	
Older than 5 years	124	79.5	65.9–88.5	20.5	11.5–34.1		123	47.9	36.2–59.8	52.1	40.2–63.8		332	98.7	95.0–99.7	1.3	0.3–5.0	
AUDIT score																		
Abstainers	347	88.2	82.6–92.1	11.8	7.9–17.4	<0.001	351	30.8	24.7–37.6	69.2	62.4–75.3	0.985	1,019	98.0	96.2–98.9	2.0	1.1–3.8	0.598
Low-risk drinkers (1–7)	108	75.2	62.8–84.5	24.8	15.5–37.2		109	31.1	20.4–44.2	68.9	55.8–79.6		326	98.9	96.9–99.6	1.1	0.4–3.1	
High-risk drinkers (8–19)	53	59.8	42.6–74.9	40.2	25.1–57.4		53	33.5	18.7–52.3	66.5	47.7–81.3		166	97.6	92.7–99.2	2.4	0.8–7.3	
Hazardous drinkers (20+)	7	81.5	41.3–96.5	18.5	3.5–58.7		7	30.1	6.0–74.5	69.9	25.5–94.0		21	74.7	47.3–90.7	25.3	9.3–52.7	
Correct knowledge of HIV and myth rejection																		
No	328	81.2	74.8–86.3	18.8	13.7–25.2	0.788	337	31.9	25.7–38.9	68.1	61.1–74.3	0.721	334	97.5	94.6–98.9	2.5	1.1–5.4	0.019
Yes	244	82.4	74.7–88.1	17.6	11.9–25.3		242	30	22.5–38.7	70	61.3–77.5		242	99.5	98.3–99.8	0.5	0.2–1.7	
Self-perceived risk																		
Low	419	84	78.7–88.2	16	11.8–21.3	0.049	425	27.2	21.8–33.3	72.8	66.7–78.2	0.008	423	98.2	95.8–99.2	1.8	0.8–4.2	0.827
High	126	73.1	60.9–82.6	26.9	17.4–39.1		128	43.9	32.8–55.6	56.1	44.4–67.2		128	98.4	95.6–99.4	1.6	0.6–4.4	
Ever had an HIV test																		
Yes	423	82.3	76.7–86.8	17.7	13.2–23.3	0.657	428	32.1	26.3–38.5	67.9	61.5–73.7	0.604	429	98.4	96.4–99.3	1.6	0.7–3.6	0.701
No	149	80.1	70.2–87.4	19.9	12.6–29.8		151	29.1	20.6–39.4	70.9	60.6–79.4		147	97.9	93.0–99.4	2.1	0.6–7.0	
Awareness of HIV status																		
Yes	324	85.9	79.8–90.4	14.1	9.6–20.2	0.038	329	33.4	26.7–40.9	66.6	59.1–73.3	0.361	331	99.2	97.8–99.7	0.8	0.3–2.2	0.052
No	248	76.7	68.9–83.0	23.3	17.0–31.1		250	28.6	21.9–36.5	71.4	63.5–78.1		245	97.2	93.3–98.8	2.8	1.2–6.7	
Final HIV status																		
Positive	47	86.5	68.8–94.9	13.5	5.1–31.2	0.500	46	52.5	34.6–69.8	47.5	30.2–65.4	0.022	46	98.3	88.4–99.8	1.7	0.2–11.6	0.771
Negative	430	81.4	75.8–85.9	18.6	14.1–24.2		439	31.1	25.6–37.2	68.9	62.8–74.4		436	98.7	97.1–99.5	1.3	0.5–2.9	

### Factors associated with risky sexual behaviors

**Figure 2:**
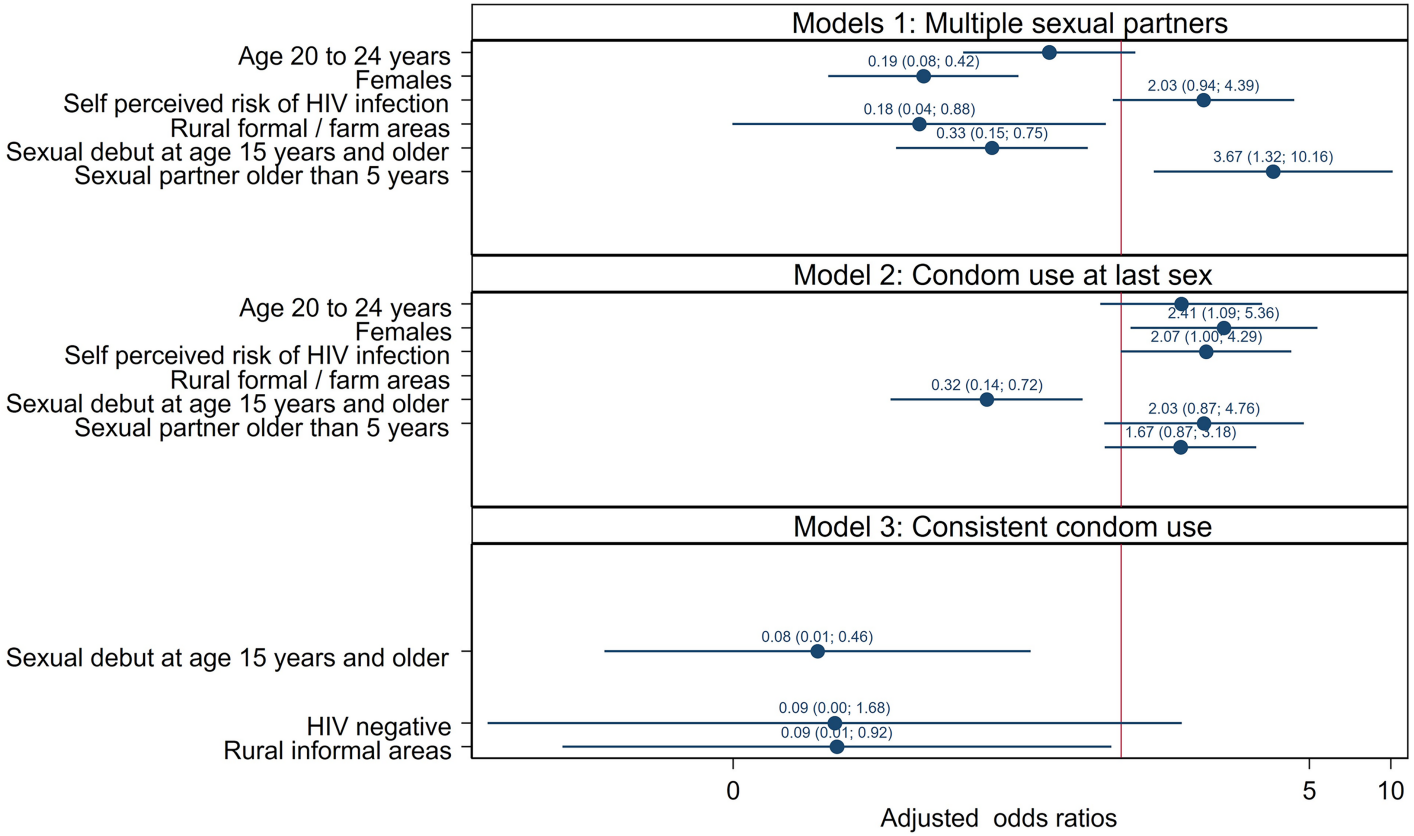
Multivariate logistic models of factors associated with having multiple sexual partners in the past year, no condom use at last sex and those with inconsistent condom use among adolescents and youth aged 12–24 years, South Africa 2017 survey.

Figure 2 shows multivariate logistic regression models of factors associated with multiple sexual partnerships, no condom use at last sex, and inconsistent condom use. The odds of reporting multiple sexual partners were significantly lower among female participants than male participants (AOR = 0.19, 95% CI: 0.08–0.42, *p* < 0.001), those residing in rural/farm areas than urban areas (AOR = 0.18, 94% CI: 0.04–0.88, *p* = 0.034), and those who reported sexual debut at age 15 years and older (AOR = 0.08, 95% CI: 0.01–0.46, *p* = 0.008)

The odds of having multiple sexual partnerships were significantly higher among those who reported sexual partners 5 years and older than within 5 years of their own age (AOR = 3.67, 95% CI: 1.32–10.16, *p* = 0.012). The odds of not using a condom at last sex were significantly higher among female participants than male participants (AOR = 2.41, 95% CI: 1.09–5.36, *p* = 0.030), and those who perceived themselves as being at high risk of HIV infection compared to their counterparts (AOR = 2.07, 95% CI: 1.00–4.29, *p* = 0.050). In addition, the odds of not using a condom at last sex were significantly lower among those who had sexual debut at age 15 years and older (AOR = 0.32, 95% CI: 0.14–0.72, *p* = 0.006). Furthermore, the odds of not using a condom consistently were significantly lower among those who had a sexual debut at age 15 years and older (AOR = 0.08, 95% CI: 0.01–0.46, *p* = 0.005), and lower among those who reside in rural formal/farm areas than urban areas (AOR = 0.09, 95% CI: 0.01–0.92, *p* = 0.043).

## Discussion

Consistent with other studies (7, 8, 12, 19), this nationally representative study found that orphaned adolescents and youth were especially at high risk of engaging in risky sexual behaviors. These included multiple sexual partnerships, not using a condom at the last sexual act, and inconsistent condom use with the most recent sexual partner. Improved understanding of predisposing factors is pivotal to addressing the needs of orphaned youth and mitigating the negative outcomes.

In the final multivariate model, the reporting of multiple sexual partners was significantly more likely among female orphans. Other studies also found a positive association between female orphans and having multiple sexual partners (8, 20). Having multiple sexual partners is one of the important behavioral risk factors for HIV (21). It has been suggested that female orphans face pressure to provide household income or assume adult obligations because of the absence of adults in the household, which could lead to having multiple sexual partners for financial support (22). These observations suggest a need for additional financial and/or economic support programs for empowering families of orphaned adolescents and youth, especially female orphans. Such interventions may include social protection strategies, such as cash transfers and nutritional support for poverty alleviation (23, 24).

The model showed that risky sexual behaviors (multiple sexual partners, no condom use at the last sex, and inconsistent condom use) were all associated with early sexual debut. This corroborates the finding that children who experience early sexual debut are likely to engage in risky sexual behaviors later in life (25, 26). Evidence shows that early sexual debut is associated with sexually transmitted infections among both orphaned male and female adolescents (27–29). The increased risk of sexually transmitted infections has been linked to a lack of condom use at first sexual encounters (29). These observations underscore the importance of raising awareness about the risk factors for early sexual initiation and highlight the need for prevention campaigns encouraging youth to delay the onset of first sex and the promotion of condom use toward risk reduction among orphaned adolescents.

Furthermore, multiple sexual partnerships and inconsistent condom use were more likely among orphans residing in urban areas. These rural–urban differences in risky sexual behaviors have been attributed to differences associated with environmental context, mainly disparities in socioeconomic and sociocultural conditions (30, 31). It has been postulated that while in urban areas individual freedom facilitates casual and higher partner change rates, people in rural areas tend to be more conservative and cultural rules toward sexual relationships are stricter (30, 31). Numerous factors could explain these differences, including poverty, the need to access material goods and money, or gift exchange in romantic relationships, especially for female adolescents in urban settings relative to rural areas (30). This suggests a need for paying attention to these differences in designing behavior modification interventions targeting orphans in urban settings.

The results showed that the reporting of no condom use at last sex was more likely among female orphans than their male counterparts. These observations have been largely attributed to gender-based power disparities related to the negotiation of condom use in sexual relationships (32). These observations underscore the need for a social paradigm shift that transforms relationships between female and male adolescents from one of inequality and dominance to equality, respect, and consideration for one another (33). This requires policy and structural interventions to overcome deeply entrenched gendered social disparities to mitigate unprotected sex, especially among orphaned adolescents and young women.

The reporting of no condom use at last sex was associated with self-perceived risk of HIV infection among orphaned adolescents and youth. This confirms the assertion that perception toward the risk of acquiring HIV depends on the history of condom use behavior and emphasizes the importance of accurately assessing an individual's risk of acquiring HIV to encourage behavior change (34–36). These results suggest a need for intensive social, behavior change and risk communication strategies in addition to increased condom availability and promotion as part of programs implemented to reduce vulnerability and/or protect orphaned adolescents and youth (37, 38).

This study has some limitations. The data used in the analysis were self-reported and therefore prone to recall and social desirability bias. The cross-section correctional nature of the study meant that causality could not be inferred, and the study was limited to ascertain the association between orphanhood and risky sexual behaviors. In addition, the small sample size and wide confidence intervals reduced the precision of estimates and power of the study. Nevertheless, the study was nationally representative and could be generalized to orphaned adolescents and youth in South Africa.

## Conclusion

The results of the study also revealed that the odds of sexual risk behaviors were higher among both male and female orphans, those who engaged in early sexual debut, those in age-disparate relationships, and those who resided in urban settings. These findings varied slightly depending on whether the outcome was multiple sexual partnerships, condom use at last sex, and inconsistent condom use. The findings suggest a need for sexual risk-reduction strategies differentiated by gender and targeted at orphans residing in urban settings. This should include sexual education interventions about delaying sexual encounters at an early age and changing HIV risk perception, prioritizing behavior modification interventions to mitigate multiple sexual partnerships and age-disparate sexual relationships, and improving condom use.

## Data Availability

The datasets presented in this study can be found in online repositories. The names of the repository/repositories and accession number(s) can be found below: The data for this manuscript are openly available on the Human Sciences Research Council institutional repository available at https://repository.hsrc.ac.za/handle/20.500.11910/15468, Archive number: SABSSM 2017 Combined, URI: http://doi.org/10.14749/1585345902.

## References

[1] Neville SE Saran I Crea TM. Parental care status and sexual risk behavior in five nationally representative surveys of sub-Saharan African nations. BMC Public Health. (2022) 22(1):59. 10.1186/s12889-021-12437-6 35012492 PMC8751264

[2] UNAIDS report on the global AIDS epidemic. New York: UNAIDS (2010).

[3] Statistics South Africa mid-year population estimates 2018. Pretoria: Stats SA (2018).

[4] Ferrand RA Corbett EL Wood R Hargrove J Ndhlovu CE Cowan FM AIDS Among older children and adolescents in Southern Africa: projecting the time course and magnitude of the epidemic. AIDS. (2009) 23(15):2039–46. 10.1097/QAD.0b013e32833016ce 19684508 PMC3408596

[5] Mejia-Pailles G Berrington A McGrath N Hosegood V. Trends in the prevalence and incidence of orphanhood in children and adolescents <20 years in rural KwaZulu-Natal South Africa, 2000–2014. PLoS One. (2020) 15(11):e0238563. 10.1371/journal.pone.0238563 33232331 PMC7685426

[6] Cluver L Orkin M Boyes M Gardner F Meinck F. Transactional sex amongst AIDS-orphaned and AIDS-affected adolescents predicted by abuse and extreme poverty. J Acquir Immune Defic Syndr. (2011) 58:336–43. 10.1097/QAI.0b013e31822f0d82 21857361

[7] Fite A Cherie A. Risky sexual behavior and its determinants among orphan and vulnerable children in Addis Ababa, Ethiopia. World J AIDS. (2016) 6(4):111–22. 10.4236/wja.2016.64015

[8] Operario D Underhill K Chuong C Cluver L. HIV infection and sexual risk behaviour among youth who have experienced orphanhood: systematic review and meta-analysis. J Int AIDS Soc. (2011) 14(1):1–11. 10.1186/1758-2652-14-25 21592368 PMC3114697

[9] Alimoradi Z Kariman N Simbar M Ahmadi F. Contributing factors to high-risk sexual behaviors among Iranian adolescent girls: a systematic review. Int J Community Based Nurs Midwifery. (2017) 5(1):2–12. 28097173 PMC5219561

[10] Fetene N Mekonnen W. The prevalence of risky sexual behaviours among youth centre reproductive health clinics users and non-users in Addis Ababa, Ethiopia: a comparative cross-sectional study. PLoS One. (2018) 13(6):e0198657. 10.1371/journal.pone.0198657 29879164 PMC5991709

[11] Rosen JG Kayeyi N Chibuye M Phiri L Namukonda ES Mbizvo MT. Sexual debut and risk behaviours among orphaned and vulnerable children in Zambia: which protective deficits shape HIV risk? Vulnerable Child Youth Stud. (2022) 17(2):130–46. 10.1080/17450128.2021.1975858 36159210 PMC9496638

[12] Toska E Gittings L Hodes R Cluver LD Govender K Chademana KE Resourcing resilience: social protection for HIV prevention amongst children and adolescents in eastern and Southern Africa. Afr J AIDS Res. (2016) 15(2):123–40. 10.2989/16085906.2016.1194299 27399042 PMC5558245

[13] Goldman PS Bakermans-Kranenburg MJ Bradford B Christopoulos A Ken PLA Cuthbert C Institutionalisation and deinstitutionalisation of children 2: policy and practice recommendations for global, national, and local actors. Lancet Child Adolesc Health. (2020) 4(8):606–33. 10.1016/S2352-4642(20)30060-2 32589873 PMC7311356

[14] Gray CL Ariely S Pence BW Whetten K. Why institutions matter: empirical data from five low- and middle-income countries indicate the critical role of institutions for orphans. In: Rus AV Parris SR Stativa E, editors. Child maltreatment in residential care: History, research, and current practice. Washington, DC: Springer International Publishing (2017), p. 379–400. 10.1007/978-3-319-57990-0_18

[15] Juma M Alaii J Bartholomew LK Askew I Van den Born B. Understanding orphan and non-orphan adolescents’ sexual risks in the context of poverty: a qualitative study in Nyanza province, Kenya. BMC Int Health Hum Rights. (2013) 13:32. 10.1186/1472-698X-13-23886019 PMC3725178

[16] Juma M Askew I Alaii J Bartholomew KL van den Borne B. Cultural practices and sexual risk behaviour among adolescent orphans and non-orphans: a qualitative study on perceptions from a community in western Kenya. BMC Public Health. (2014) 14:84. 10.1186/1471-2458-14-84 24467940 PMC3912900

[17] Simbayi L Zuma K Zungu N Moyo S Marinda E Jooste S South African National HIV prevalence, incidence, behaviour and communication survey, 2017. Cape Town: HSRC Press (2019).

[18] Stats SA. Spatial metadata. Pretoria: Stats SA (2011).

[19] Embleton L Nyandat J Ayuku D Sang E Kamanda A Ayaya S Sexual behavior among orphaned adolescents in western Kenya: a comparison of institutional- and family-based care settings. J Adolesc Health. (2017) 17(4):417–24. 10.1016/j.jadohealth.2016.11.015 PMC5389113 28110864

[20] Birdthistle IJ Floyd S Machingura A Mudziwapasi N Gregson S Glynn JR. From affected to infected? Orphanhood and HIV risk among female adolescents in urban Zimbabwe. AIDS. (2008) 22(6):759–66. 10.1097/QAD.0b013e3282f4cac7 18356606

[21] Bingenheimer JB. Men’s multiple sexual partnerships in 15 sub-Saharan African countries: sociodemographic patterns and implications. Stud Fam Plann. (2010) 41(1):1–17. 10.1111/j.1728-4465.2010.00220.x 21151707 PMC2998893

[22] Magnusson BM Crandall A Evans K. Early sexual debut and risky sex in young adults: the role of low self-control. BMC Public Health. (2019) 19:1483. 10.1186/s12889-019-7734-9 31703650 PMC6839049

[23] Cluver LD Orkin FM Boyes ME Sherr L. Cash plus care: social protection cumulatively mitigates HIV-risk behaviour among adolescents in South Africa. AIDS. (2014) 28(Suppl 3):S389–97. 10.1097/QAD.0000000000000340 24991912

[24] Cluver LD Hodes RJ Sherr L Orkin FM Meinck F Lim Ah Ken P Social protection: potential for improving HIV outcomes among adolescents. J Int AIDS Soc. (2015) 18(Suppl 6):20260. 10.7448/IAS.18.7.20260 26639115 PMC4670837

[25] Beegle K Krutikova S. Adult mortality and children's transition into marriage. Demogr Res. (2008) 14(4):1551–74. 10.4054/DemRes.2008.19.42

[26] Conduct Problems Prevention Research Group. Trajectories of risk for early sexual activity and early substance use in the fast track prevention program. Prev Sci. (2014) 15(Suppl 1(01)):S33–46. 10.1007/s11121-012-0328-8 23417666 PMC3883936

[27] Thurman TR Brown L Richter L Maharaj P Magnani R. Sexual risk behaviour among South African adolescents: is orphan status a factor? AIDS Behav. (2006) 10(6):627–35. 10.1007/s10461-006-9104-8 16838071

[28] Fatusi A Wang W. Multiple sexual partnership mediates the association between early sexual debut and sexually transmitted infection among adolescent and young adult males in Nigeria. Eur J Contracept Rep Health Care. (2009) 14(2):134–43. 10.1080/13625180802601110 19340709

[29] Shafii T Stovel K Holmes K. Association between condom use at sexual debut and subsequent sexual trajectories: a longitudinal study using biomarkers. Am J Public Health. (2007) 97(6):1090–5. 10.2105/AJPH.2005.068437 17463388 PMC1874201

[30] Thior I Rowley E Mavhu W Kruse-Levy N Messner L Falconer-Stout ZJ Urban-rural disparity in sociodemographic characteristics and sexual behaviours of HIV-positive adolescent girls and young women and their perspectives on their male sexual partners: across-sectional study in Zimbabwe. PLoS One. (2020) 15(4):e0230823. 10.1371/journal.pone.0230823 32324764 PMC7179911

[31] Wiafe S Mihan A Davison CM. Neighbourhood-level influences and adolescent health risk behaviours in rural and urban sub-Saharan Africa: a systematic review. Int J Environ Res Public Health. (2021) 18(14):7637. 10.3390/ijerph18147637 34300089 PMC8305046

[32] Muldoon KA Duff PK Birungi J Ngolobe MH Min JE King R Decisions, decisions: the importance of condom use decision making among HIV sero-discordant couples in a prospective cohort study in Uganda. Sex Transm Infect. (2014) 90(5):408–12. 10.1136/sextrans-2013-051326 24695989

[33] Conroy AA Ruark A Tan JY. Re-conceptualising gender and power relations for sexual and reproductive health: contrasting narratives of tradition, unity, and rights. Cult Health Sex. (2020) 22(sup1):48–64. 10.1080/13691058.2019.1666428 31633456 PMC7170748

[34] Muchiria E Odimegwua C De Weta N. HIV risk perception, and consistency in condom use among adolescents and young adults in urban Cape Town, South Africa: a cumulative risk analysis. S Afr J Infect Dis. (2017) 32(3):105–10. 10.1080/23120053.2017.1332800

[35] Schaefer R Thomas R Maswera R Kadzura N Nyamukapa C Gregson S. Relationships between changes in HIV risk perception and condom use in East Zimbabwe 2003–2013: population-based longitudinal analyses. BMC Public Health. (2020) 20(1):756. 10.1186/s12889-020-08815-1 32448365 PMC7245904

[36] Haine RA Ayers TS Sandler IN Wolchik SA. Evidence-based practices for parentally bereaved children and their families. Prof Psychol Res Pract. (2008) 39(2):113. 10.1037/0735-7028.39.2.113 PMC2888143 20585468

[37] Lombe M Ochumbo A. Sub-Saharan Africa’s orphan crisis: challenges and opportunities. Int Soc Work. (2008) 51(5):682–98. 10.1177/0020872808093345

[38] UNAIDS. AIDS in Africa: three scenarios to 2025 (2005). Available at: http://www.unaids.org/unaids_resources/Homepage/images/AIDSScenarios/AIDSscenarios-2025_report_en.pdf (Accessed November 26, 2006).

